# Predicting the Combined Effects of Multiple Stressors and Stress Adaptation in *Gammarus pulex*

**DOI:** 10.1021/acs.est.4c02014

**Published:** 2024-07-10

**Authors:** Naeem Shahid, Ayesha Siddique, Matthias Liess

**Affiliations:** † System-Ecotoxicology, Helmholtz Centre for Environmental Research − UFZ, Permoserstraße 15, 04318 Leipzig, Germany; ‡ Department of Evolutionary Ecology and Environmental Toxicology, Goethe University Frankfurt, 60629 Frankfurt am Main, Germany; § Institute for Environmental Research (Biology V), RWTH Aachen University, Worringerweg 1, 52074 Aachen, Germany

**Keywords:** combined effects, mixture toxicity, fitness costs, genetic adaptation, synergism

## Abstract

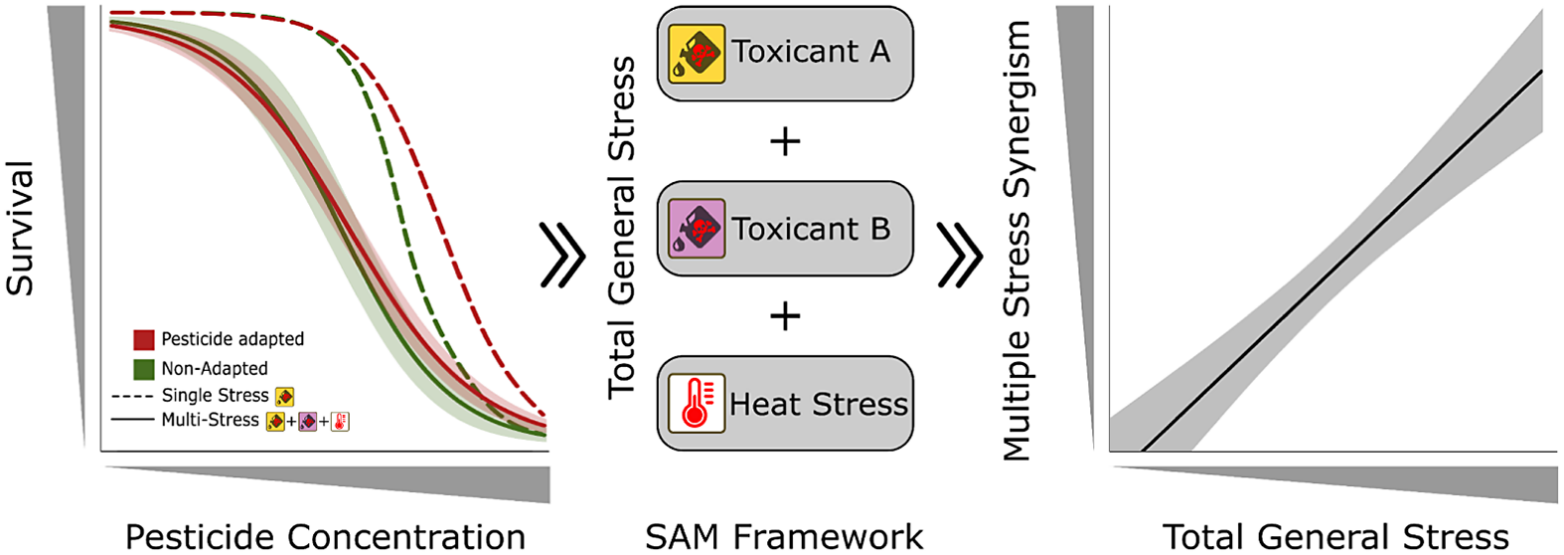

Global change confronts organisms with multiple stressors causing nonadditive effects. Persistent stress, however, leads to adaptation and related trade-offs. The question arises: How can the resulting effects of these contradictory processes be predicted? Here we show that *Gammarus pulex* from agricultural streams were more tolerant to clothianidin (mean EC_50_ 148 μg/L) than populations from reference streams (mean EC_50_ 67 μg/L). We assume that this increased tolerance results from a combination of physiological acclimation, epigenetic effects, and genetic evolution, termed as adaptation. Further, joint exposure to pesticide mixture and temperature stress led to synergistic interactions of all three stressors. However, these combined effects were significantly stronger in adapted populations as shown by the model deviation ratio (MDR) of 4, compared to reference populations (MDR = 2.7). The pesticide adaptation reduced the General-Stress capacity of adapted individuals, and the related trade-off process increased vulnerability to combined stress. Overall, synergistic interactions were stronger with increasing total stress and could be well predicted by the stress addition model (SAM). In contrast, traditional models such as concentration addition (CA) and effect addition (EA) substantially underestimated the combined effects. We conclude that several, even very disparate stress factors, including population adaptations to stress, can act synergistically. The strong synergistic potential underscores the critical importance of correctly predicting multiple stresses for risk assessment.

## Abstract 2

This is the initial study that quantitatively predicts the synergism of pesticide mixtures, environmental stress, and the fitness cost of pesticides under multistress conditions.

## Introduction

The planetary boundaries for climate change, chemical pollution, land-use change, and nutrients exceed the safe limits for biodiversity conservation.^1,2^ However, this exceedance may indicate even a greater problem when stressor interactions are considered. So far, the impact assessment does not explicitly consider the potential interactions between stressors, instead it focuses on individual stressors in isolation. This is mainly because the interactions between different stressors are complex and cannot be predicted as no general framework is existing to calculate these interactions. The usefulness of the Planetary Boundaries framework for understanding the global risks of current environmental stressors would be greatly enhanced if stress interactions could be predicted.

The combined effects of multiple stressors can be additive (equal to the sum of individual stressors), antagonistic (less than additive), or synergistic (more than additive). These interactions are determined in relation to the applied null model. If stressors are not interacting, combined effects can be predicted based on single-stressor effects.^3,4^ Such effects can be predicted following the classic assumptions of concentration addition (CA; Bliss^5^) for toxicants having similar modes of action and effect addition (EA; Loewe and Muischnek^6^) for stressors with different modes of action. Both models have extensively been employed to predict additive effects of mixtures.^7−10^ However, in the case of interactive stressors, joint effects deviate from the conventional null models, indicating antagonism or synergism, which requires more complex models for reliable prediction.^11,12^

Pesticides are often applied as mixtures or sequential applications, leading to their co-occurrence in freshwaters, especially after rainfall events.^13−16^ In the past decade, neonicotinoids were the most commonly applied class of insecticides in the study area and worldwide.^17−19^ Azole fungicides have also frequently been used, often detected in European surface waters,^13,20^ and are known to interact synergistically with different insecticides.^21−24^ Further, organisms in the field experience sub- or supra-optimal conditions and are forced to cope with complex environmental stress.^25^

Under climate change scenario, extreme temperature is one of the most relevant stressors that can further enhance the effects of pesticides.^26,27^ Increased temperature may pose physiological stress to aquatic organisms by increasing metabolic rate associated with the mechanisms of thermal tolerance.^28,29^ Even though several studies have shown that environmental stress may interact with toxicants,^30−33^ it remains a question how adaptation to pesticides influences the interaction between pesticide mixtures and environmental stressors.^34^ Adaptation depends on trade-offs between the benefits of immediate stress responses and their long-term fitness costs. Numerous studies have reported fitness costs of pesticide adaptation in aquatic and terrestrial organisms.^35−38^ Adaptation to a single stressor can increase the impact of multiple stressors in aquatic invertebrates.^39−41^ Thus, the fitness cost may emerge as a stressor, particularly under unfavorable conditions within the ecological context. To enable efficient ecosystem management, we need to determine the ecological relevance of each of these stressors. This can only be achieved if we have tools at hand that can predict the effects of multiple stressors.

We aimed to reveal the combined effects of a frequently detected insecticide clothianidin and an azole fungicide prochloraz in combination with warming—a most relevant environmental stressor under climate change. Further, we investigated how pesticide adaptation shapes multiple stress–response relationships. We hypothesized that agricultural populations may possess advantages in the face of pesticide mixture compared to reference populations. Further, stressors with different modes of action, such as insecticide, fungicide, and elevated temperature, could potentially interact, with stronger effects expected in adapted populations. We also hypothesized that the individual stress induced by each stressor can be quantified by SAM, and the synergism increases with increasing total stress of the interacting stressors. For this, we investigated populations of the widespread aquatic crustacean *Gammarus pulex* from contaminated and reference streams and exposed them to a mixture of pesticides and temperature stress. Furthermore, we predicted combined impacts using traditional models to distinguish possible interactions of toxicant mixtures (i.e., concentration addition (CA; Bliss^5^) and effect addition (EA; Loewe and Muischnek^6^) and stress addition model (SAM; Liess et al.^12^)) designed to quantify synergistic interactions between independent stressors. With this approach, we performed the first study to predict the interactions of pesticide mixtures, environmental stress, and the fitness cost of pesticide adaptation, which may act as a stressor under multistress conditions. Due to its high topical relevance, we expect that the approach presented here will be the starting point for a fundamental expansion of our understanding of the effect of multiple stressors.

## Materials and Methods

### Sampling of Test Organisms and Characterization of Pesticide Pollution in the Field

In the present study, we investigated the sensitivity of *G. pulex* against clothianidin and prochloraz at different temperature regimes. Individuals were collected from 12 sites: 8 from high to low pesticide-contaminated agricultural streams and 4 from close to uncontaminated streams located in central Germany (Figure S1). From each selected stream, we collected approximately 1000 *G. pulex* individuals with a size ranging between 6 and 10 mm, using a 25 × 25 cm kick-net with a 500 μm mesh size. Individuals were gently captured using a pipet and transferred into aerated and cooled plastic boxes filled with streamwater and transported to the laboratory. Subsequently, organisms were acclimatized to three different temperatures (16, 19, and 22 °C) over a period of 10 days in ADaM^42^ (artificial daphnia medium), with 350 individuals from each population. The investigation was carried out during spring (April-May) 2021, before the peak period for pesticide application.

This investigation was conducted as part of the nationwide small stream monitoring (kgM) project,^13^ and data on community structure and pesticide pollution were also obtained from this data set published on PANGAEA.^43^ The toxic pressure of pesticide pollution was quantified by analyzing the event-driven runoff samples and grab samples in 2018, 2019, and 2021 during the peak application of pesticides (April–July). Rain-event-triggered water samples were collected using automated (MAXX TP5, Rangendingen, Germany) and bottle samplers (EDS; Liess and Von Der Ohe^44^) to capture runoff-induced peak exposures after rainfall.^45^ Run-off events raise the water level of streams that trigger the samplers to capture peak concentrations. Automated samplers take 5 mL water every 5 min from the stream for 3.3 h, yielding 200 mL of water samples. Collected samples were kept at 4 °C in samplers until they were transported to the laboratory within 48 h. Grab samples were collected regularly after every 3 weeks, which is similar to the monitoring practices suggested by the Water Framework Directive (WFD).

A wide range of pesticides (108) and urban toxicants (257) were analyzed. All pesticides were quantified using liquid chromatography MS/MS, whereas urban toxicants were quantified using liquid chromatography–high-resolution mass spectrometry (LC–HRMS) as mentioned earlier.^13^

### Calculation of Toxicant Exposure

To estimate pesticide-induced toxic pressure of stream sections, measured concentrations were transformed into toxic units (TUs) by dividing them with their respective acute LC_50_ or EC_50_ for the standard test organisms.^46^ For each pesticide, we used either *D. magna* or *C. riparius*, selecting the most sensitive of the two species.^13^ To obtain a representation of the toxic pressure of a site, we used the pesticide providing maximum toxic unit (TU_max_) (eq 1).^44^
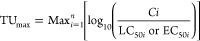
1where TU_max_ is the highest value of the toxic unit, *Ci* is the detected concentration of the pesticide (μg L^–1^), and LC_50i_ or EC_50i_ is the respective acute median lethal/effective concentration (μg L^–1^) for the most sensitive reference organism. For the calculation of TU_max_, LC_50_ or EC_50_ values of the most sensitive species was used and obtained from the Pesticide Properties Database (PPDB)^47^ and Ecotoxicology Database System.^48^ To show a comprehensive, time-integrated picture of pesticide exposure, we aggregated the TU (toxic unit) values for each site by integrating both current and previous data.^13,49^

### Characterizing the Ecological Effects of Pesticides

We used a bioindicator SPEAR_pesticides_ to quantify the long-term impact of pesticides on macroinvertebrate community structure in the field.^44^ The SPEAR index quantifies the toxic pressure of pesticides by classifying macroinvertebrates as vulnerable and nonvulnerable taxa based on different ecological traits. We calculated SPEAR values using the Indicate software (Version 1.1.1; https://systemecology.de/indicate/). To increase reliability and reduce data variance, we aggregated the SPEAR values for each site by incorporating both current and previous data.^13,49^

### Acute Toxicity Experiments

The acute toxicity experiments were conducted following the rapid testing approach and adopted OECD guidelines for testing chemicals.^50,51^ We selected clothianidin and azole fungicide prochloraz for the pesticide mixtures. To identify the ecological consequence of global warming, we applied 19 and 22 °C as the temperature stress to study the mechanistic effect of elevated temperature, in addition to a reference temperature of 16 °C, which is in the range of the optimum temperature for *G.pulex*. The temperatures are within field-relevant ranges, as indicated by recent national monitoring data. Approximately 40% of the streams showed water temperatures exceeding 19 °C (75th quantile of all measuring points), with 10% of all sites exceeding 21 °C from April to June (PANGAEA).^43^

In the present study, we used a neonicotinoid insecticide clothianidin as a primary chemical stressor to generate a dose response curve and to characterize the sensitivity of *G. pulex*. To investigate the toxicological interactions of pesticide mixture, we introduced a fungicide prochloraz as an additional chemical stressor at 3 different concentrations. To prepare the clothianidin stock solution, we used granulated powder (weight ratio 1:1) from DANTOP (Spiess-Urania Chemical GmbH, Germany). Forty milligram of the powder was diluted in 0.5 L of deionized water, resulting in a final concentration of 40 mg clothianidin per liter. The mixture was then thoroughly mixed overnight on a magnetic stirrer. However, prochloraz (CAS 67747-09-5, purity: 98.6%) stock solution was prepared using DMSO as a solvent. Stock solutions were further diluted in Artificial Daphnia Medium to prepare the required test concentrations. The maximal solvent concentration in treatments was 0.001% [vol/vol], which is approximately 200 times below the No Observed Effect Concentration (NOEC) established for *Daphnia magna*(52) and ensures that the concentration used in our experiments does not induce any adverse biological effects. The DMSO concentration was also below the solvent limit recommended by OECD test guidelines.^53^

**Figure 1:**
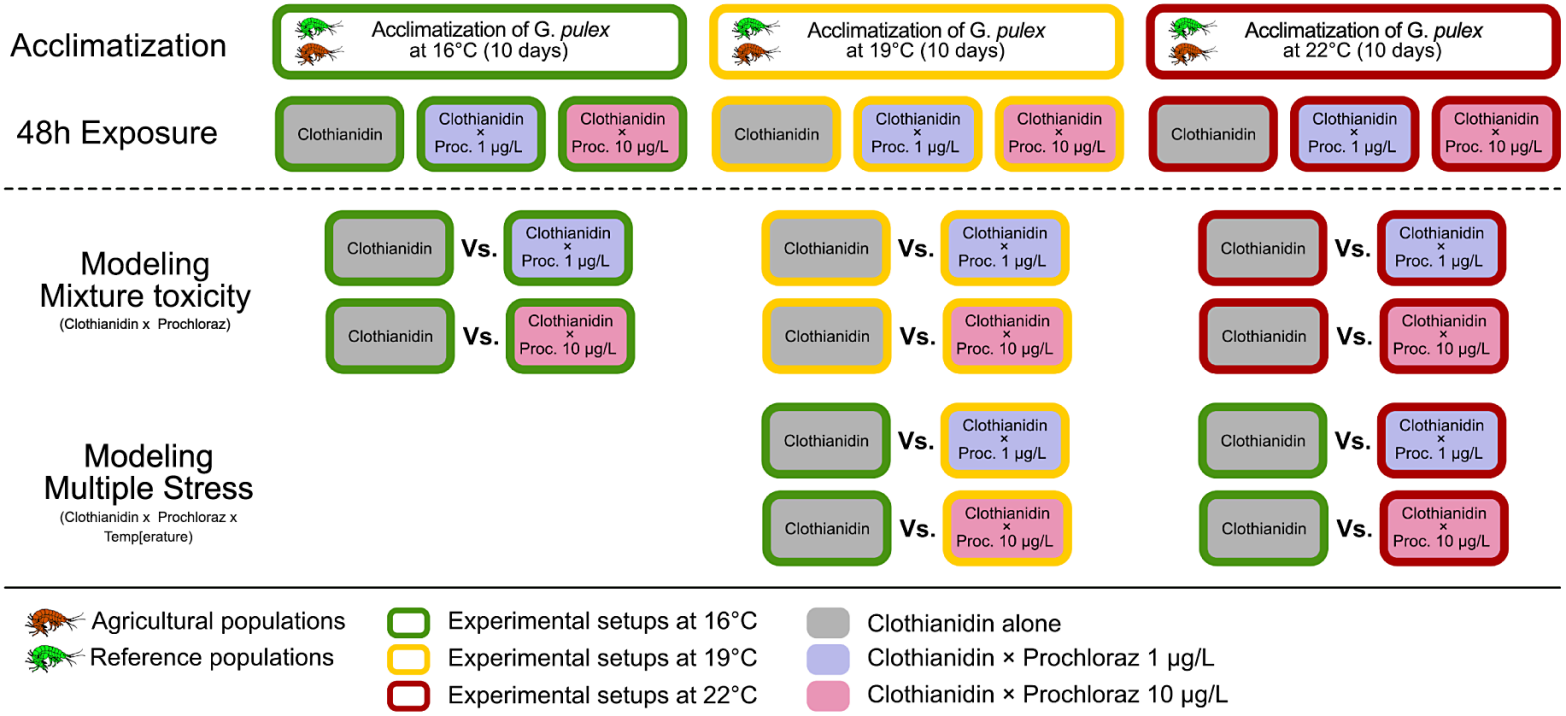
Overview of the experimental design. *Gammarus pulex* was collected from agricultural and reference streams. Both populations were acclimatized to different temperatures (green open rectangle, 16; yellow open rectangle, 19; red open rectangle, 22 °C) for 10 days and exposed to a range of clothianidin (0 to 1000 μg/L) for 48 h under nine different conditions: three prochloraz treatments (gray filled rectangle, 0; blue filled rectangle, 1; magenta filled rectangle, 10 μg/L) × three temperatures (green open rectangle, 16; yellow open rectangle, 19; red open rectangle, 22 °C). Subsequently, the interaction between both pesticides was predicted at different temperatures. For multiple stress, the EC_50_ values of clothianidin under elevated temperate were compared with the control at 16 °C.

For mixture toxicity and multiple stress, we set up a full factorial design with nine clothianidin concentrations (0, 0.01, 0.1, 1, 10, 100, 215, 465, and 1000 μg/L) × three prochloraz treatments (0, 1, and 10 μg/L) × three temperatures (16, 19, and 22 °C). Before pesticide exposure, 350 individuals from each population were acclimatized to three different temperatures (16, 19, and 22 °C) for 10 days. For each treatment, we exposed 12 individuals from each population (4 individuals per tea bag, diameter 6 cm). The exposure was done in 5 L beakers containing 3 L of medium. The beakers were placed in climate chambers with a 16:8 light–dark cycle and continuous aeration, and the immobility was recorded for 48 h. If organisms did not move their bodies within 20 s, even after probing with a rod, they were considered immobile. Fanning of gills and antenna did not count for body movement. To quantify the exposure concentrations of clothianidin and prochloraz, we collected 250 mL of the stock and test concentrations and analyzed them using GC–MS/MS by SGS GmbH, Hamburg, Germany. Actual concentrations recovered from the samples were within acceptable boundaries (±10%) to the nominal concentrations. An overview of the experimental design is provided in Figure 1.

### Data Analyses and Prediction of Combined Effects

For the data analyses and graphical representations, we used RStudio version 2023.06.1 for Windows^54^ and the basic R version 4.3.1 for Windows.^55^ To compare clothianidin tolerance of gammarid populations under different stress conditions, we calculated EC_50_ (median effective concentration) from the toxicity experiments using the five-parameter log–logistic model.^56^ We compared clothianidin tolerance represented by median effective concentration (EC_50_), toxic pressure (TU), and ecological status (SPEAR index) of agricultural and reference streams using a two-sample *t* test (data with equal variances) and Welch’s *t* test (data with nonequal variances). For the association between different factors such as toxic pressure (TU_max_) and the change in macroinvertebrate community composition or the clothianidin tolerance, we applied linear regressions. Before analyses, we confirmed the normal distribution and homoscedasticity of residuals and ln(*x*) transformed EC_50_ values to obtain a normal distribution.

To gain better understanding of the interaction between various stressors, we predicted the combined effects of two chemical stressors (clothianidin and prochloraz) under “Mixture Toxicity” and explored the combined effects of chemical stressors and elevated temperature under “Multiple Stress” (see Figure 1). For both analyses, survival per treatment was averaged for agricultural and reference groups. Under “Mixture Toxicity,” we tested our hypothesis that agricultural populations tolerate a pesticide mixture better than reference populations when a toxic mixture is applied. We investigated interactions between clothianidin and prochloraz across various temperature regimes. For this purpose, we compared the EC_50_ of clothianidin in the presence of prochloraz (i.e., 1 and 10 μg/L) at 16, 19, and 22 °C with their respective controls (control at 0 μg/L prochloraz, as illustrated in Figure 1). Under “Multiple Stress,” we investigated the combined effects of both pesticides and suboptimal temperatures in agricultural and reference populations, as illustrated in Figure 1. For this purpose, we compared the EC_50_ values of all setups under higher temperature regimes (i.e., 19 and 22 °C) with their respective controls at 16 °C, without prochloraz (representing the best-case scenario). Furthermore, we applied paired sample *t* test to compare the synergistic interactions between stressors among agricultural and reference populations. In all the comparisons of synergism, we used EA-based MDR values.

To quantify the individual stress induced by each stressor, such as prochloraz and elevated temperature, we employed dose–response curves of reference populations exposed to clothianidin alone and in the presence of the respective additional stressor. We then compared the immobility rates in two scenarios: (i) clothianidin alone and (ii) clothianidin + additional stressor, specifically around the EC_50_ of clothianidin alone. The additional immobility caused by the second stressor was subsequently converted into General-Stress using the SAM. A linear regression was applied to examine the relationship between the sum of the total stress and synergism expressed in terms of model deviation ratio (MDR).

To predict cumulative response to mixture toxicity and multiple stressors (as illustrated in Figure 1), two conventional approaches for mixture toxicity such as concentration addition (CA; Loewe and Muischnek^6^) and effect addition (EA; Bliss^5^), and SAM (Liess et al.^12^) were employed. In comparison to CA and EA, the SAM model was designed to predict the cumulative impacts of toxicants and environmental stressors.^12^ These models were further compared for their predictive accuracy.

According to the EA model, the combined effect was calculated by eq 2.^5^
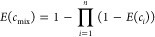
2where *E*(*c*_mix_) is the joint effect of *E*(*c*_*i*_) stressors.

For the concentration addition model (CA), the sum of the toxic units corresponding to the mixture components was calculated by eq 2.^6^
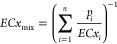
3where *ECx*_mix_ is the sum of concentrations of toxicants present in the mixture, *pi* represents the relative fraction of toxicant *i*, and *ECx_i_* is the concentration of the toxicant *i* posing × % effect.

According to the SAM, stress-dependent survival was calculated by eq 3.^12^
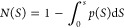
4where *N*(*S*) = 1 (100% survival) for the general stress *S* = 0 and *N*(*S*) = 0 (0% survival) for the general stress *S* ≥ 1. The total general stress “*S*” was calculated as the sum of general stress levels *S_i_* of all independently acting stressors (for details, see Liess et al^12^). 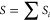
5

For the prediction of combined effects (i.e., EC_50_), we applied EA, CA, and SAM models using a web-based application (Indicate, version 2.2.1; http://www.systemecology.eu/indicate/). For the predictive accuracy of these models, we divided predicted EC_50_ values by the observed EC_50_ values and calculated the MDR. The MDR closer to 1 (MDR ≈ 1) indicates a higher accuracy of the model in quantifying the combined effects of multiple stressors.

We used EA as a null model for the combined effects. MDR values <0.5 indicated antagonistic response from exposure to a toxicant mixture and values >1 (more than additive) indicated synergism. If the MDR values are between 1 and 2 (>1 <2), we consider it as weak synergism; otherwise, if MDR is greater than 2, it is considered strong synergism.

## Results

### Pesticide Exposure and Ecological Effects

In total, 365 targeted substances were analyzed in the streamwater samples. In terms of toxic units (log TU_max_, see Materials and Methods), pesticide contamination ranged from −3.1 to −0.8 TU in agricultural streams, with a mean of −2.1 TU, which has been shown to cause ecological effects. In contrast, reference streams were contaminated only to a minor extent (log TU_max_: – 4.8 to −3.6, mean: −4.2), which is considered safe for the ecosystem.

**Figure 2:**
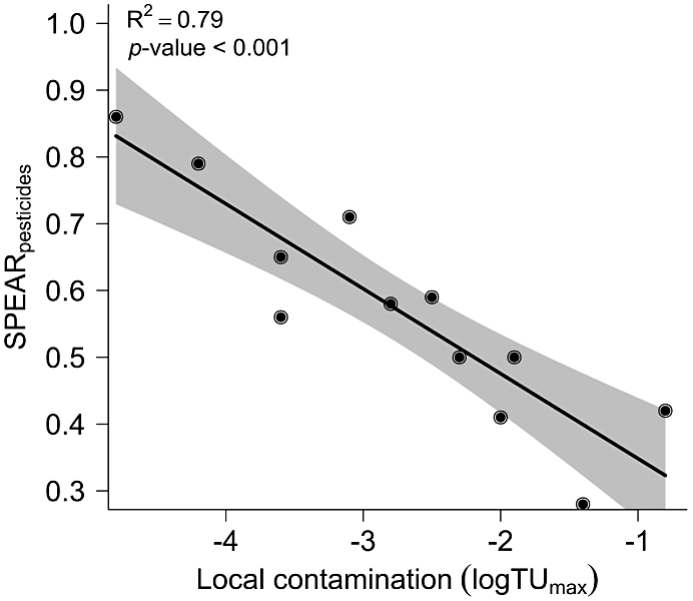
Effects of pesticide contamination on macroinvertebrate community structure quantified with the biological indicator SPEAR: Local pesticide contamination changes the macroinvertebrate community structure (linear regression, adjusted *R*^2^ = 0.79, *F* = 41.86, residual df = 10, *p* < 0.001). Shaded areas represent 95% confidence intervals.

We quantified the ecological impacts of pesticide contamination by the change in macroinvertebrate community composition using the SPEAR_pesticides_ bioindicator and observed lower SPEAR values (i.e., 0.28 to 0.71; mean 0.50) in agricultural streams, indicating a reduced proportion of species vulnerable to pesticides. In contrast, reference streams showed higher SPEAR values, indicating an increased proportion of species vulnerable to pesticides (i.e., 0.56 to 0.86; and mean 0.71). Accordingly, the macroinvertebrate community structure significantly depended on local pesticide contamination (log TU_max_; adjusted *R*^2^ = 0.79, *p* < 0.001; Figure 2). To establish more robust and reliable association, we used aggregated data on pesticide contamination and macroinvertebrate community structure from multiple years at each site. Reference streams (log TU_max_ < −3.5) were characterized by significantly higher SPEAR values in comparison to pesticide-contaminated agricultural streams (Wilcoxon’s rank sum test, *W* = 28, *p* = 0.05).

**Figure 3:**
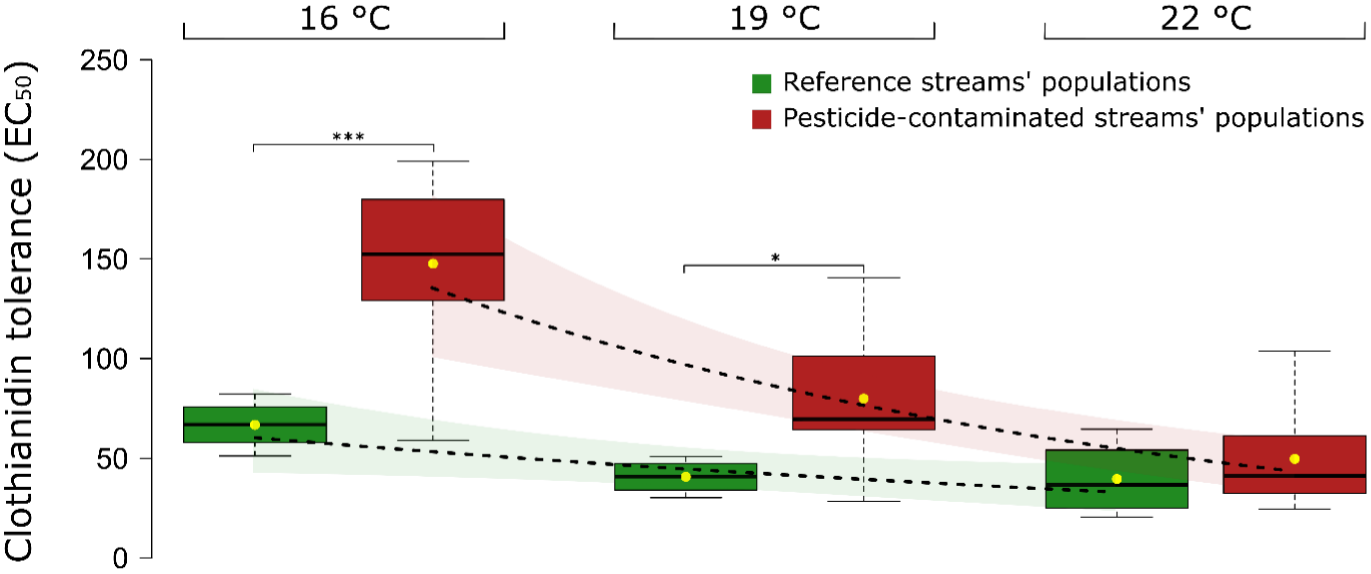
Pesticide tolerance of populations from eight contaminated and four reference streams quantified with their clothianidin tolerance (EC_50_) under different warming conditions: EC_50_ of *G. pulex* collected from control (green) and agricultural streams (red) after exposure (48 h) to clothianidin under different temperature regimes (16, 19, and 22 °C). The lower and upper boundaries of the box represent the 25th and 75th percentile, the horizontal line denotes the median, and the whiskers correspond to the lowest and highest values. Dashed lines represent fitted regressions with confidence intervals displayed by shaded areas. The significance level is displayed as * for *p* < 0.05, ** for *p* < 0.01, and *** for *p* < 0.001.

*G. pulex* from agricultural streams showed higher tolerance (EC_50_) to pesticides than those from reference streams. Laboratory investigations revealed that at reference temperature (16 °C), agricultural populations were 2.2-fold more tolerant to clothianidin as compared to reference populations (EC_50_ values: reference = 67 μg/L, agricultural = 148 μg/L, *t* = −4.7284, df = 8.9215, and *p* < 0.001; Figure 3). The tolerance of both, the adapted and the nonadapted populations, significantly decreased with increase in temperature.

Agricultural populations exposed at 19 and 22 °C were, respectively, 1.9- (EC_50_: 79 μg/L) and 3.0-fold (EC_50_: 50 μg/L) less tolerant to clothianidin as compared to the reference temperature of 16 °C (148 μg/L). However, the reference populations showed less decrease in tolerance with increase in temperature (reference populations: slope = −4.5, agricultural populations: slope = −16.8, *p* < 0.001). The average EC_50_ decreased by 1.6-fold (EC_50_: 41 μg/L) at 19 °C and 1.7-fold (EC_50_: 39 μg/L) at 22 °C. Thus, the difference in tolerance between the two groups also decreased with increase in temperature—from 2.2-fold at 16 °C (*p* < 0.001) to 1.9-fold at 19 °C (*p* < 0.05) and finally 1.3-fold at 22 °C, which was not significantly different anymore (*p* > 0.5) (Figure 3).

### Interaction Between Clothianidin and Prochloraz

In the form of mixture, both very low prochloraz exposure setups slightly increased the sensitivity of *G. pulex* to clothianidin (Figure S2, *R*^2^=0.1, *p* < 0.001). To test our hypothesis that agricultural populations tolerate a pesticide mixture better than reference populations, we compared the toxicity of clothianidin at 0, 1, and 10 μg/L of prochloraz under three temperature regimes (16, 19, and 22 °C). The EC_50_ values were compared with their respective controls (as shown in Figure 1). In both populations, prochloraz showed weak synergistic interaction (i.e., MDR > 1 < 2) with clothianidin even at the highest concentration (10 μg/L, Table 1). However, the combined effects of this pesticide mixture were significantly stronger in reference populations (paired sample *t* test; *p* < 0.05).

**Table 1:** Prediction of Joint Effects of Neonicotinoid Clothianidin Alone and in Combination with a Fungicide Prochloraz under Different Temperature Regimes^a^

					MDR		
	Temp°C	Prochloraz(μg/L)	Observed EC_50_^b^(μg/L)	Predicted EC_**50**_^c^(μg/L)	CA	EA	SAM
Mixture toxicity (interaction between clothianidin and prochloraz at different temperatures)^d^							
Reference populations	16	0	66.82				
	16	1	58.20	66.82	1.15	1.15	1^e^
	16	10	45.02	66.82	1.48	1.48	1^e^
	19	0	40.32				
	19	1	31.10	40.32	1.30	1.30	1.11
	19	10	25.41	40.32	1.59	1.59	1.11
	22	0	38.21				
	22	1	27.96	38.21	1.37	1.36	1.19
	22	10	24.64	38.21	1.55	1.55	1.12
Agricultural populations	16	0	145.34				
	16	1	175.49	145.34	0.83	0.85	0.70
	16	10	117.44	145.34	1.24	1.27	0.84
	19	0	75.84				
	19	1	67.15	75.84	1.13	1.13	0.95
	19	10	60.26	75.84	1.26	1.30	0.85
	22	0	50.75				
	22	1	33.31	50.75	1.52	1.54	1.27
	22	10	36.99	50.75	1.37	1.37	0.90
Multiple stressors (interaction between clothianidin, prochloraz, and temperature stress)^f^							
Reference populations	*Control of 16 °C*		66.82				
	19	0	40.39	66.46	1.65	1.65	1^e^
	19	1	31.12	66.46	**2.15**	**2.14**	1.02
	19	10	25.40	66.28	**2.63**	**2.61**	1.03
	22	0	38.24	66.33	1.75	1.73	1^e^
	22	1	27.97	66.21	**2.39**	**2.37**	0.98
	22	10	24.67	66.46	**2.71**	**2.69**	0.91
Agricultural populations	*Control of 16 °C*		145.34				
	19	0	75.83	148.69	1.92	1.96	0.98
	19	1	67.06	146.61	**2.17**	**2.19**	0.89
	19	10	60.19	152.45	**2.41**	**2.53**	0.79
	22	0	50.70	149.65	**2.87**	**2.95**	1.24
	22	1	36.55	150.47	**3.98**	**4.12**	1.38
	22	10^e^	36.27	149.65	**4.01**	**4.13**	1.10

a Strong synergistic interactions of stressors (MDR > 2) are indicated in bold.

b The observed EC50s for clothianidin are based on the average survival of the respective populations (i.e., agricultural and reference) and calculated using a five-parameter log-logistic model.

c The predicted EC50 values are calculated by the CA as a null model.

d Under mixture toxicity, we compared the EC_50_ of clothianidin for prochloraz concentrations (i.e., 1 and μg/L) at 16, 19, and 22 °C in relation to their respective controls without prochloraz.

e We employed these dose–response curves to quantify the individual stress induced by each stressor, and therefore, the MDR value for SAM is 1 (see Materials and Methods).

f For multiple stressors, we compared all treatments of prochloraz under higher temperature regimes (i.e., 19 and 22 °C) with respective control of agricultural and reference populations at 16 °C and without prochloraz (best case).

### Interaction Between Multiple Stressors

**Figure 4:**
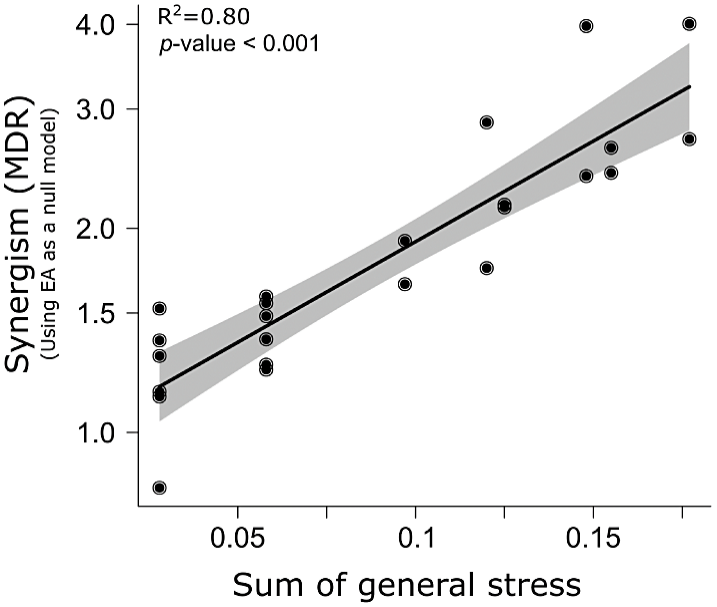
Relationship between total General-Stress and strength of synergistic effects: combined stress of multiple stressors including prochloraz, suboptimal temperature, and fitness cost of pesticide adaptation significantly increased the clothianidin sensitivity expressed by synergism with effect addition as null model (linear regression, adjusted *R*^2^ = 0.80, *F* = 93.28, residual df = 22, *p* < 0.001). Shaded areas represent 95% confidence intervals.

For the combined effect of multiple stressors, we compared the EC_50_ values of all setups under higher temperature regimes (i.e., 19 and 22 °C) with their respective controls at 16 °C, without prochloraz (representing the best-case scenario). In reference populations, temperature stress caused weak synergistic interaction (MDR > 1 < 2) with clothianidin (Table 1). However, in agricultural populations, elevated temperature increased the sensitivity of individuals to clothianidin much stronger than for reference populations, indicated by MDR values of 1.92 and 2.87 at 19 and 22 °C, respectively. Further, the combination of prochloraz and temperature stress notably increased clothianidin sensitivity of individuals from both agricultural and reference populations (Table 1). The interaction of multiple stress—expressed in terms of MDR values—was significantly stronger in agricultural populations (paired sample *t* test; *p* < 0.05) and caused up to 2-fold higher synergism of multiple stressors (using EA as a null model) in agricultural populations (Table 1). To identify the association between synergism and General-Stress, we used the General-Stress approach of the SAM framework to calculate the individual stress posed by different stressors, including clothianidin, prochloraz, and elevated temperature, and added them according to SAM to quantify the total General-Stress (see Materials and Methods). Overall, synergism increased with increase in total stress of all the stressors (Null model EA: Figure 4, *R*^2^ = 0.80).

### Predictive Accuracy of Models

We used concentration addition (CA), effect addition (EA), and the SAM to predict the combined effects of (i) pesticide mixtures and (ii) multiple stress including elevated temperature. In both cases, SAM showed considerably superior predictive accuracy for combined effects compared to the additive models (CA and EA), as indicated by SAM’s MDR values closer to 1.0 (Table 1) and the modeled curves (Figures S3 and S4). However, CA and EA considerably underestimated the combined effects of all stressors, particularly for pesticide-adapted populations, with underestimations of up to 4-fold (Table 1 and Figures S3 and S4).

## Discussion

Here we found that all the stressors with different modes of action, including clothianidin, prochloraz, and suboptimal temperature, interacted synergistically. Additionally, the fitness costs associated with pesticide adaptation acted as an additional stressor under multiple stress conditions (Table 1). Moreover, all the stressors with different modes of action, including clothianidin, prochloraz, and suboptimal temperature, can be added to calculate the overall General-Stress and predict their synergistic effects. This is substantially extending most studies on multiple stressors that focus on binary stressors.^30,57−59^ In our present study, all the stressors synergistically interacted according to their individual strengths, contrasting the notion that the stronger stressor overrides the effect of weaker stressors.^60,61^

Clothianidin is a neonicotinoid insecticide that affects nicotinic acetylcholine receptors,^62^ whereas azole fungicides inhibit a wider range of cytochrome P450s^63^ and are known to interact synergistically.^21^ Our results show that the agricultural populations were significantly more tolerant to clothianidin alone and also the pesticide mixture as compared to the reference populations. This higher tolerance develops due to pesticide adaptation resulting from prior exposure in the field.^40,49^ Transient pesticide exposure may result in physiological acclimation.^64,65^ In contrast, genetic adaptation is considered to prevail particularly under consistent and regular exposure over multiple generations,^65^ which is likely the case for *G. pulex* in agricultural streams.^66,67^ This is also supported by the observation that populations from agricultural streams are characterized by specific alleles occurring generally in contaminated streams.^68^ We therefore assume that this increased pesticide tolerance in agricultural populations might be a combination of physiological acclimation, epigenetic effects, and genetic evolution.

However, both the agricultural and reference populations showed synergistic responses to the joint stress of pesticides and temperature. Elevated temperature may pose physiological stress to aquatic organisms by increasing metabolic rate associated with the mechanisms of thermal tolerance,^28,29^ but the nature of interactions with chemical stressors are not consistent. In the present study, synergism was significantly stronger in agricultural populations adapted to pesticide pollution (Table 1). In general, environmental stressors with different modes of action may interact synergistically with chemical stressors.^12,32,69^ For example, Delnat et al.^70^ observed a synergistic interaction of high variation in daily temperature with a mixture of chlorpyrifos and *Bacillus thuringiensis* toward *Culex pipiens*. Similarly, Macaulay et al.^71^ reported synergistic combined effects of the heat wave and a neonicotinoid insecticide imidacloprid on mayfly nymphs. However, we used constant temperatures aimed at isolating the mechanistic effects of temperature on pesticide toxicity in a controlled setting. Although this approach has limitations in terms of realism, it offers a clearer baseline for understanding the combined stress of complex multiple stressors. Liess et al.^12^ also identified, in a meta-analysis, that increasing stress from environmental stressors systematically increases the vulnerability of various organisms to toxicant stress. Contrary to this, some investigations observed little or no effects,^72^ or even extreme antagonistic effects.^57,73,74^ However, these investigations did not focus on stress adaptation. Recently, Siddique et al.^40^ and Heim et al.^75^ reported increased sensitivity of pesticide-resistant populations to the temperature stress. It is suggested that the mechanisms of tolerance development cause energetic constraints, which may result in trade-offs between different fitness-related functions. Therefore, the stronger synergistic response of pesticide-adapted populations might be attributed to the lack of a plastic response, suggesting higher costs to maintain pesticide tolerance.^76^

A critical challenge in predicting the combined effects of multiple stresses is to establish a “common currency” to quantify and integrate different stressors.^77^ The SAM assumes that each organism has a “General-Stress capacity” toward all types of specific stressors.^12^ This concept enables us to transform different stressors into General-Stress levels. Accordingly, here we calculated the individual sum of stress posed by different stressors, including clothianidin, prochloraz, and elevated temperature, and added them to quantify total General-Stress. Each stressor reduced the common stress capacity of individuals. Thus, the synergism of multiple stressors was getting stronger with increasing total General-Stress (Figure 4). So far, SAM has been employed to assess the interaction between toxicants and environmental stressors,^12,78^ and toxicant mixtures.^78^

Overall, conventional multistress models (CA and EA) underestimated the combined impacts of clothianidin and prochloraz under higher temperature regimes (multiple-stress conditions; Table 1 and Figure S3 and S4). These results are crucial because they underscore the limitation of EA to predict the combined effects of independent stressors, as attempted here. It is not surprising for interacting multiple stressors because CA and EA assume concentration- and effect-related additive effects^5,6^ and can only predict the combined effects of mixtures if the synergistic or antagonistic interactions between chemicals are absent. However, these approaches have frequently been used to formally identify whether the interaction type is antagonistic, additive, or synergistic. Instead, SAM predicted the combined multiple stressor impacts better than CA and EA even in pesticide-adapted populations (Table 1 and Figures S3 and S4). SAM presumes that the combined impact of stressors with different modes of action can be calculated by adding up individual effects transformed to the General-Stress and then compared with the General-Stress capacity of the individuals within a population.^12^ Obviously, this approach is highly successful in predicting the combined effects of different stressors. As a next step, it will be relevant to identify whether the synergy observed in the laboratory is likely to manifest in the aquatic environment also under natural conditions requires further studies. Evidence of the synergistic effect of toxicant mixtures in the field was found by recording the effect of herbicides in agricultural waters.^79^ Also, the synergistic effect of warming and pesticides has already been identified in agricultural waters.^26^ It is therefore very likely that the relationships and mechanism presented here are also relevant on the ecosystem level.

Accordingly, the current study is an important step toward ecological realism in risk assessment by revealing interactions of pesticide mixtures, environmental stress, and the fitness costs of pesticide adaptation. Our results show that multiple stressors such as clothianidin, prochloraz, elevated temperature, and pesticide adaptation interact synergistically, and therefore, pesticide-adapted gammarid populations become more vulnerable to global warming. Although predicting the combined impacts of multiple stressors was a great challenge so far, we successfully used the SAM to calculate total General-Stress and showed that the synergism increases with increase in total stress of the interacting stressors.

## Supporting Information Available

Location of the sampling sites in central Germany that cover a wide range of pesticide pollution from noncontaminated to highly contaminated streams (Figure S1); tolerance to clothianidin decreased with increase in the concentration of prochloraz (Figure S2); survival of *Gammarus pulex* exposed to a neonicotinoid insecticide clothianidin and an azole fungicide prochloraz at 19 °C (Figure S3); survival of *Gammarus pulex* exposed to a neonicotinoid insecticide clothianidin and an azole fungicide prochloraz at 22 °C (Figure S4); information of the investigated streams including physicochemical parameters in terms of TU_max_ values and the composition of the macroinvertebrate community structure expressed as SPEAR_pesticides_ (Table S1) (PDF)
