# Supplementary material for: Predicting the Combined Effects of Multiple Stressors and Stress Adaptation in Gammarus pulex
Source: Environ Sci Technol. 2024 Jul 10;58(29):12899–908. doi: 10.1021/acs.est.4c02014 (PMC11270985; doi:10.1021/acs.est.4c02014)
Supplement: Supplementary file 1 — es4c02014_si_001.pdf [file es4c02014_si_001.pdf]

Supporting Information for:

Predicting the Combined Effects of Multiple Stressors and Stress Adaptation in *Gammarus pulex*

Author affiliations:

1. Author: Naeem Shahid[1, 2, *] (naeem.shahid@ufz.de, phone: 0341 235 1495)
2. Author: Ayesha Siddique[1, 3] (ayesha.siddique@ufz.de, phone: 0341 235 1495)
3. Author: Matthias Liess[1, 3] (matthias.liess@systemecology.de, phone: +49 341 235 1578)

[1]System-Ecotoxicology, Helmholtz Centre for Environmental Research – UFZ, Permoserstraße 15, 04318 Leipzig, Germany

[2]Department of Evolutionary Ecology and Environmental Toxicology, Goethe University Frankfurt, 60629 Frankfurt am Main, Germany

[3]Institute for Environmental Research (Biology V), RWTH Aachen University, Worringerweg 1, 52074 Aachen, Germany

[*]Corresponding author

This file contains 6 pages including 5 figures and 1 table

Table of contents

Figures S1 to S4 .... S2
Table S1 ....S6

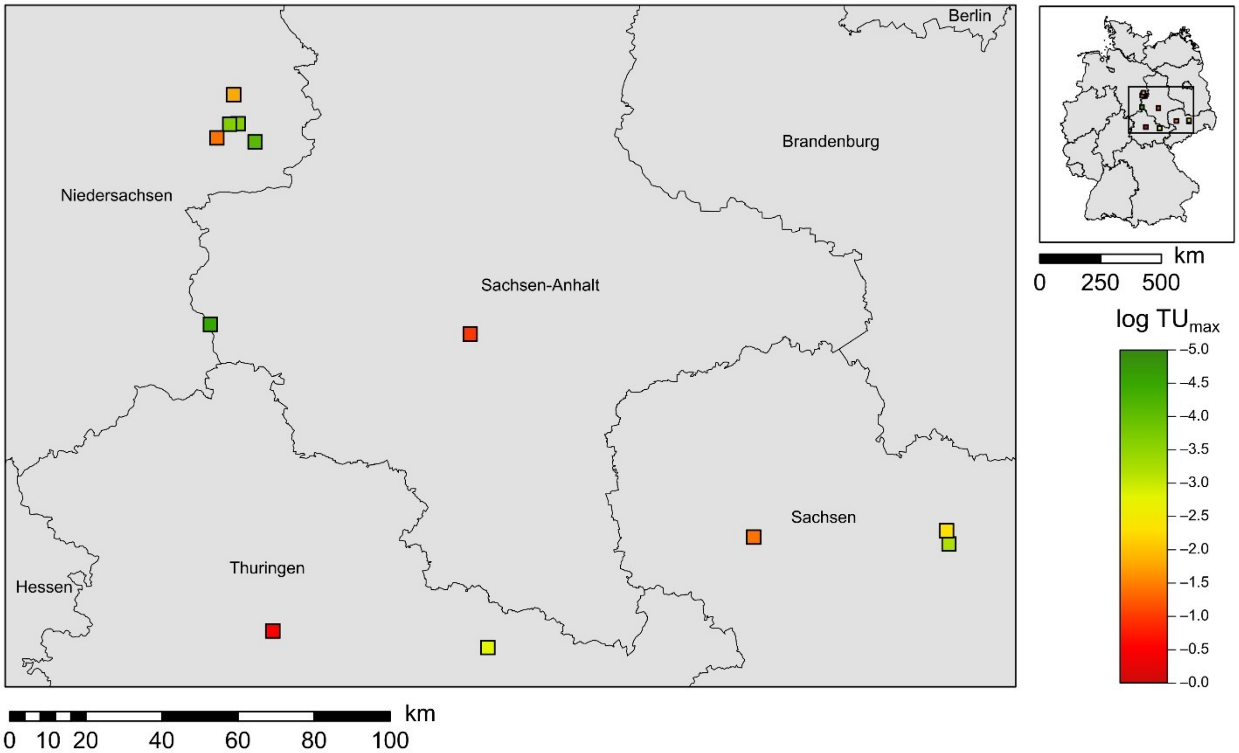

Figure S1: Location of the sampling sites in central Germany that cover a wide range of pesticide pollution from non-contaminated to highly contaminated streams. Square shapes represent sampling sites and are coloured according to the local contamination (log TU_{max}) ranging from low (green) to high contamination (red).

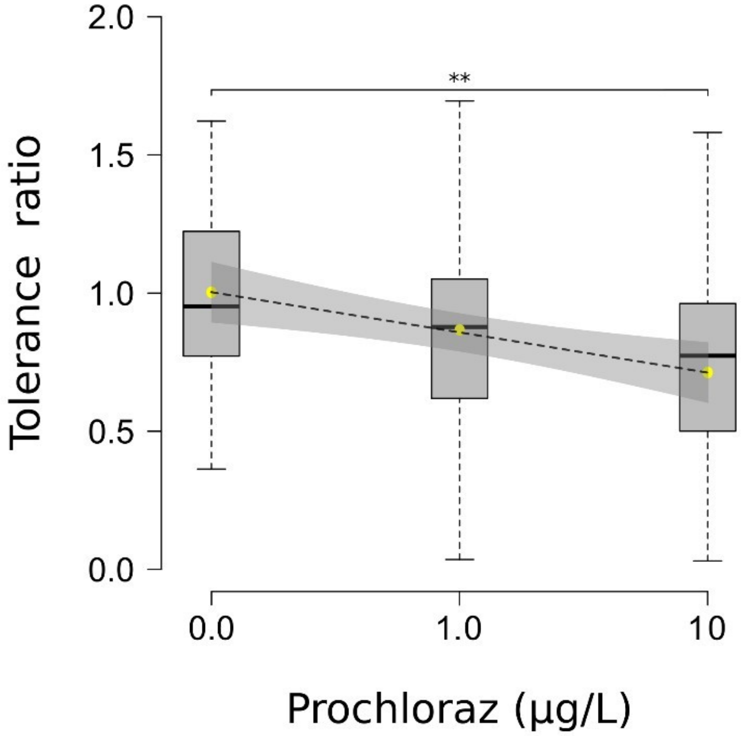

Figure S2: Tolerance to clothianidin decreased with increasing concentration of prochloraz. The tolerance ratio was calculated by dividing the EC_{50} of each population by the average EC_{50} of the respective controls without prochloraz. The lower and upper boundaries of the box represent the 25th and 75th percentile, the horizontal line denotes the median, and the whiskers correspond to the lowest and highest values. Dashed lines represent fitted regression with confidence intervals displayed by shaded areas. The significance level is displayed as "**" for $p < 0.01$.

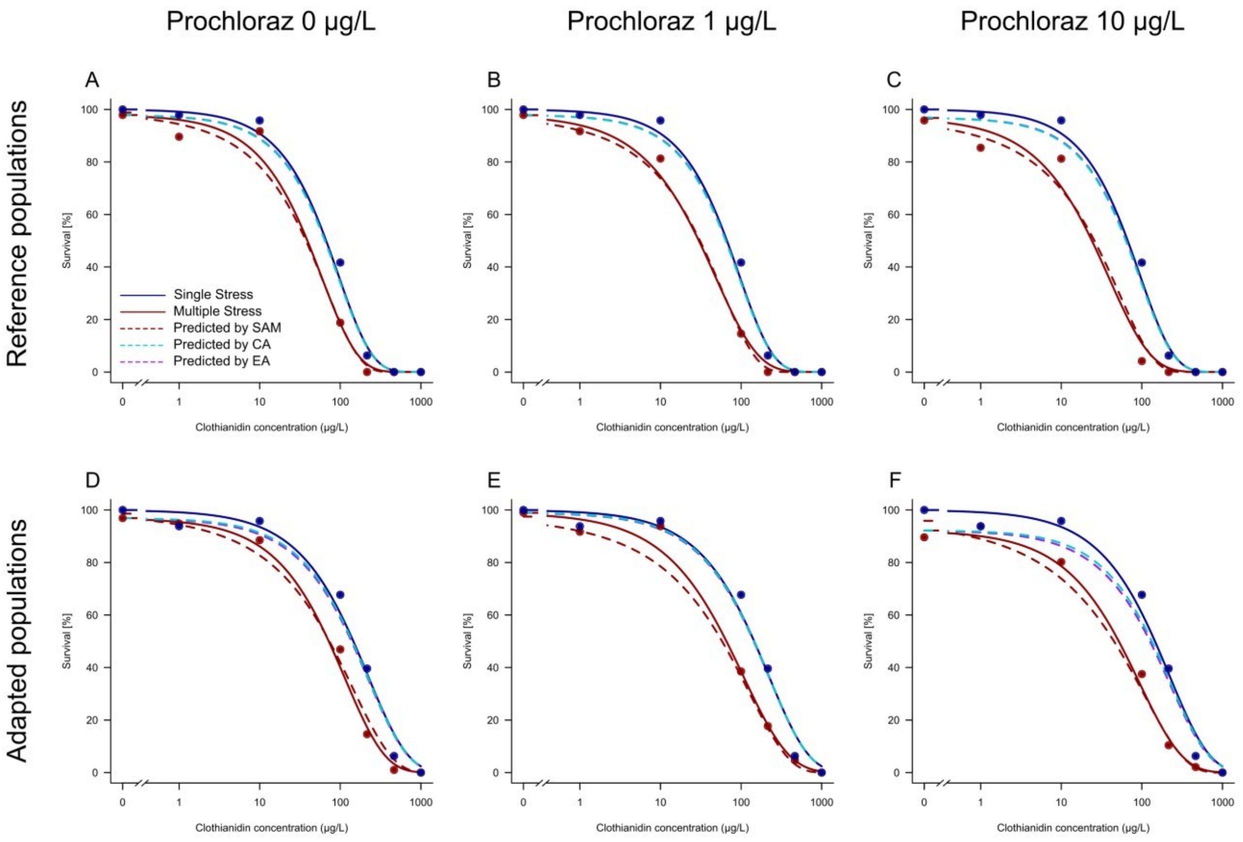

Figure S3. Survival of *Gammarus pulex* exposed to a neonicotinoid insecticide clothianidin and an azole fungicide prochloraz at 19°C. Dose-response relationships are displayed for reference (A-C) and agricultural (D-F) populations - without additional stress at 16°C (blue points, solid line) and in combination with different prochloraz concentrations (A, D; 0µg/L, B, E; 1µg/L and C, F; 10µg/L) and elevated temperature (19°C) as additional stressors (red points, solid line). Data points represent the average survival of the populations from the respective group. The red dashed line represents the modelled concentration-response relationship under additional stress using the Stress Addition Model (SAM); whereas, violet and cyan dashed lines represent the EA and CA models respectively. According to the SAM framework, the individual stress of each stressor was: prochloraz 1µg/L: 0.028, prochloraz 10µg/L: 0.058 and 19°C: 0.097.

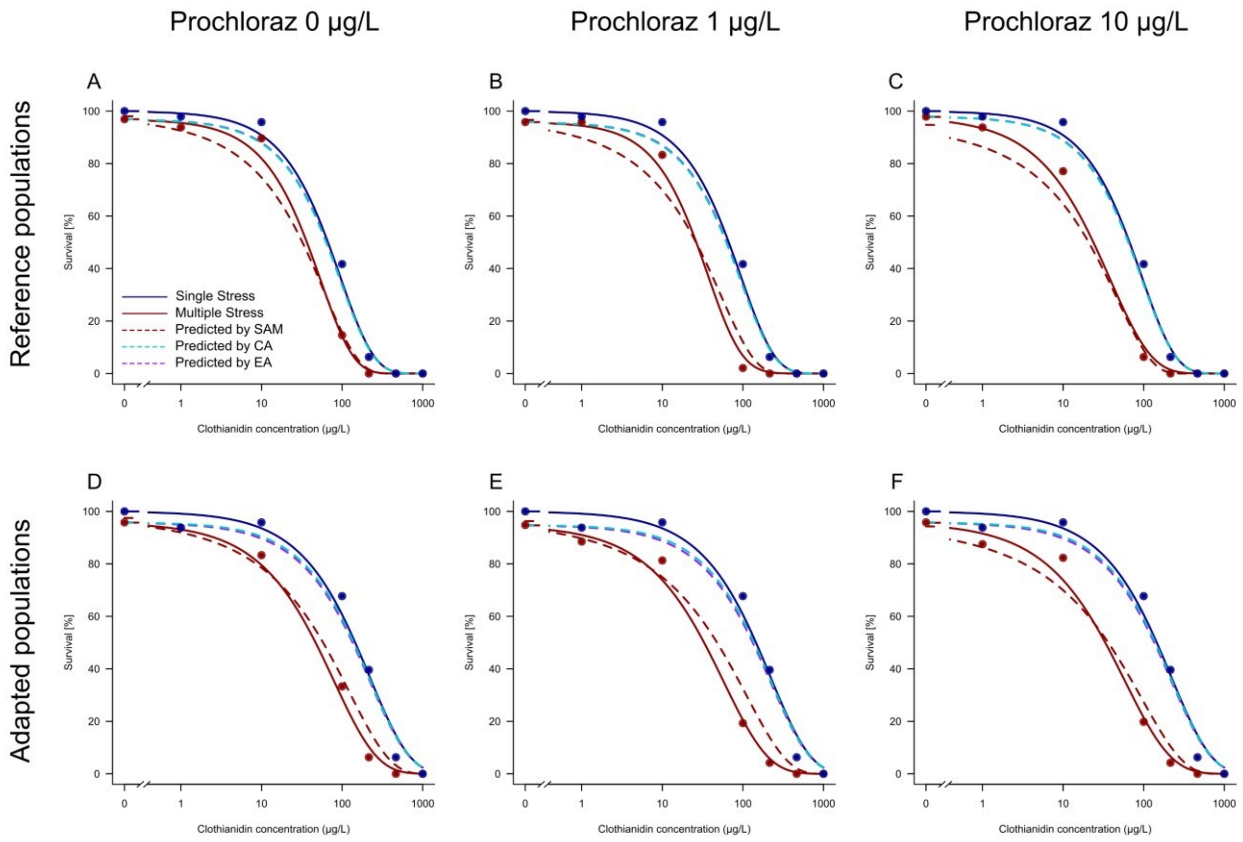

Figure S4. Survival of *Gammarus pulex* exposed to a neonicotinoid insecticide clothianidin and an azole fungicide prochloraz at 22°C. Dose-response relationships are displayed for reference (A-C) and agricultural (D-F) populations - without additional stress at 16°C (blue points, solid line) and in combination with different prochloraz concentrations (A, D; 0µg/L, B, E; 1µg/L and C, F; 10µg/L) and elevated temperature (22°C) as additional stressors (red points, solid line). Data points represent the average survival of the populations from the respective group. The red dashed line represents the modelled concentration-response relationship under additional stress using the Stress Addition Model (SAM); whereas, violet and cyan dashed lines represent the EA and CA models respectively. According to the SAM framework, the individual stress of each stressor was: prochloraz 1µg/L: 0.028, prochloraz 10µg/L: 0.058 and 22°C: 0.12.

Table S1: Information of the investigated streams including physicochemical parameters such as water temperature (°C), water level (cm), dissolved oxygen (DO) and pH, water toxicity in terms of TU_{max} values, and the composition of the macroinvertebrate community structure expressed as $SPEAR_{pesticides}$.

Sites ID	Temperature [°C]	Water level (cm)	DO [mg/L]	pH	*Temperature [°C]	Toxic Unit (TU_{max})	SPEAR
Agri 1	9.3	19	13.56	8.45	19.6	-1.9	0.5
Agri 2	5.6	19	12.7	8.07	19.4	-3.1	0.71
Agri 3	7.6	34	12.82	7.75	NA	-2.8	0.58
Agri 4	5.3	24	11.92	7.54	18.1	-2.5	0.59
Agri 5	6.8	36	14.42	7.89	19.5	-2	0.41
Agri 6	5.8	10	13.93	8.61	17.2	-1.4	0.28
Agri 7	7.2	25	13	8.23	17.4	-2.3	0.5
Agri 8	6.4	30	15.88	8.34	16.1	-0.8	0.42
Non-Agri 1	7.1	12	7.59	7.77	18.6	-4.2	0.79
Non-Agri 2	7.1	20	11.7	7.89	20.3	-4.8	0.86
Non-Agri 3	4.5	18	12.66	7.88	17.1	-3.6	0.56
Non-Agri 4	6.9	18	12.11	8.1	17.1	-3.6	0.65

Temperature, Water level, DO and pH are measured at the time of sampling, whereas, *Temperature is the 75th quantile of all measuring points in the data series measured by probes (April to June), and missing values were replaced by average of similar sites.
